# The neuronal insulin sensitizer dicholine succinate reduces stress-induced depressive traits and memory deficit: possible role of insulin-like growth factor 2

**DOI:** 10.1186/1471-2202-13-110

**Published:** 2012-09-18

**Authors:** Brandon H Cline, Harry WM Steinbusch, Dmitry Malin, Alexander V Revishchin, Galia V Pavlova, Raymond Cespuglio, Tatyana Strekalova

**Affiliations:** 1 Interdisciplinary Center for Neurosciences, Heidelberg University, and Institute for Neuroanatomy, University Clinic Heidelberg, Im Neuenheimer Feld 307, 69120, Heidelberg, Germany; 2 School for Mental Health and Neuroscience, Department of Neuroscience, Maastricht University, Universiteitssingel 40, NL, 6229 ER, Maastricht, Netherlands; 3 University of Wisconsin, Carbon Cancer Center, WIMR 3016, 1111 Highland Ave, Madison, WI, 53705, USA; 4 Institute of General Pathology and Pathophysiology, Russian Academy of Medical Sciences, Baltiyskaya str. 8, 125315, Moscow, Russia; 5 Institute of Gene Biology of Russian Academy of Sciences, 34/5 Vavilov str, Moscow, 119334, Russia; 6 Claude Bernard University, Lyon1, Faculty of Medicine, EA 4170, Av. Rockefeller 8, 69373, Lyon, CEDEX 08, France

**Keywords:** Dicholine succinate, Insulin-like receptor, Insulin growth factor 2, Hippocampus, Stress-induced anhedonia, Mouse

## Abstract

**Background:**

A number of epidemiological studies have established a link between insulin resistance and the prevalence of depression. The occurrence of depression was found to precede the onset of diabetes and was hypothesized to be associated with inherited inter-related insufficiency of the peripheral and central insulin receptors. Recently, dicholine succinate, a sensitizer of the neuronal insulin receptor, was shown to stimulate insulin-dependent H_2_O_2_ production of the mitochondrial respiratory chain leading to an enhancement of insulin receptor autophosphorylation in neurons. As such, this mechanism can be a novel target for the elevation of insulin signaling.

**Results:**

Administration of DS (25 mg/kg/day, intraperitoneal) in CD1 mice for 7 days prior to the onset of stress procedure, diminished manifestations of anhedonia defined in a sucrose test and behavioral despair in the forced swim test. Treatment with dicholine succinate reduced the anxiety scores of stressed mice in the dark/light box paradigm, precluded stress-induced decreases of long-term contextual memory in the step-down avoidance test and hippocampal gene expression of IGF2.

**Conclusions:**

Our data suggest that dicholine succinate has an antidepressant-like effect, which might be mediated via the up-regulation of hippocampal expression of IGF2, and implicate the neuronal insulin receptor in the pathogenesis of stress-induced depressive syndrome.

## Background

Recent epidemiological studies have established a link between diabetes and the prevalence of depression [1,2]. The presence of depressive symptoms is documented in 12.8–29% of males and 23.8–30.5% of females with newly diagnosed diabetes [3,4]. A positive relationship between insulin resistance and the severity of depressive symptoms has been identified in cross-sectional studies [5,6]. Depression in patients with diabetes can result from the chronic psychological and medical conditions associated with the disease [7,8]; however, the occurrence of depression was found to precede the onset of diabetes which, apart from the behavioral factors and changes in eating habits often accompanying depression, might be associated with inherited inter-related insufficiency of the peripheral and central insulin receptors [9,10].

**Figure 1:**
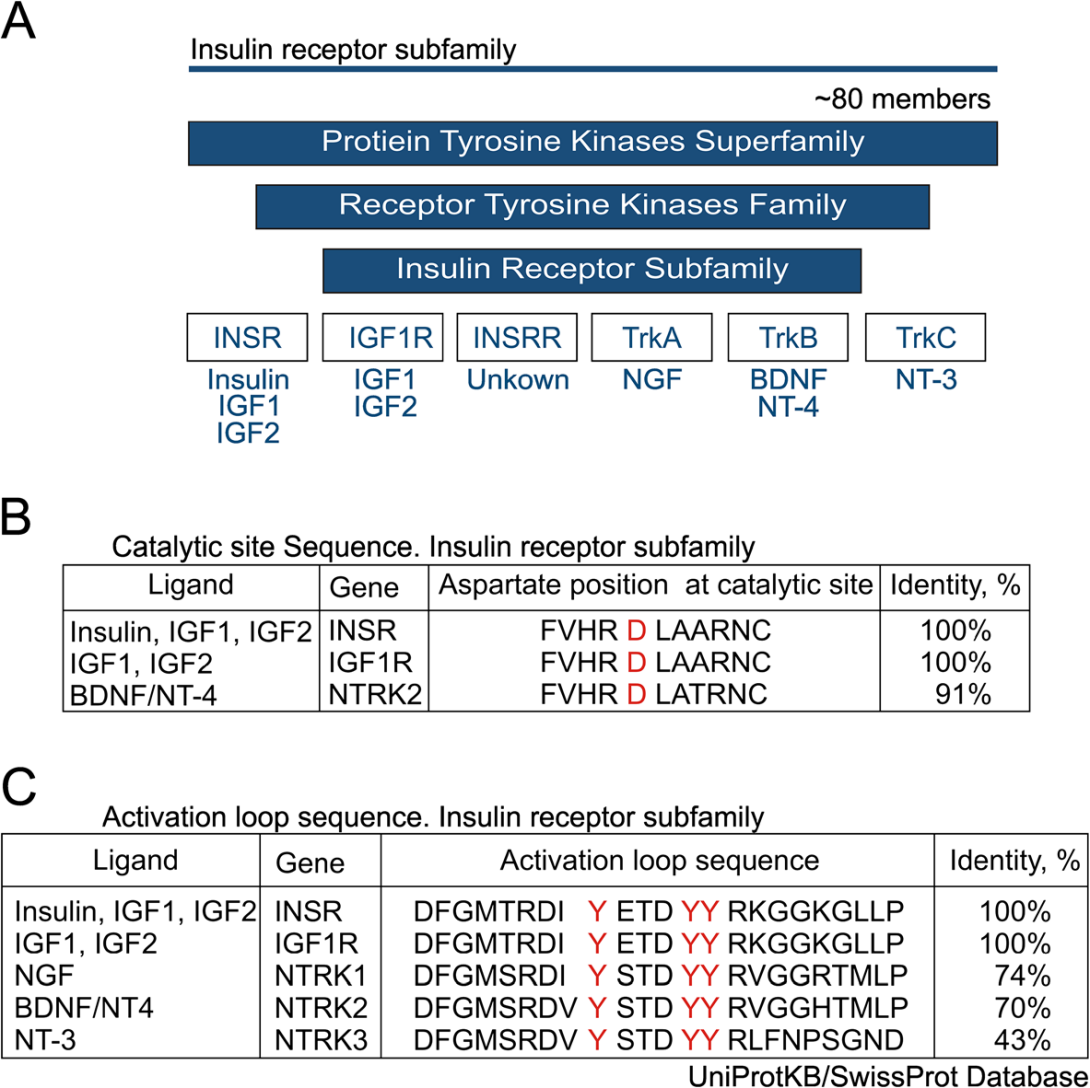
**Insulin receptor subfamily of receptor tyrosine kinase family.** (Only functionally important areas of interaction shown). (**A**) The neuronal insulin receptor (INSR), insulin-like growth factor 1 receptor (IGF1R), insulin receptor-related protein (INSRR), and receptors to neurotrophins NGF (NTRK1), BDNF and NT-4 (NTRK2), and NT-3 (NTRK3) belong to an insulin receptor subfamily of receptor tyrosine kinases whose members are well documented to regulate cell survival and differentiation and play a role in synaptic plasticity. (**B**) Analysis with the UniProt/KB Swiss-Prot data bank reveals high structural homology between the catalytic site sequences of the insulin receptor, insulin-like growth factor 1 receptor, and NTRK2 (TrkB) receptor. (**C**) Structure comparison with the UniProt/KB Swiss-Prot data bank suggests high structural homology of the activation loop sequences of the insulin receptor, insulin-like growth factor 1 receptor, NTRK1, NTRK2 (TrkB), and NTRK3 receptors.

The neuronal insulin receptors belong to an insulin receptor subfamily of receptor tyrosine kinases (Figure 1A) and many of these receptors have been shown to be involved in multiple mechanisms of synaptic plasticity as well as differentiation and cell survival [11-13]. Analysis using the UniProt/KB Swiss-Prot data bank revealed high structural homology between the catalytic sites and the activation loops of the insulin receptor and TrkB (Figures 1B, C); it is well known that TrkB manifests a role in the stress response [14-16]. The neuronal insulin receptor is involved in the control of synaptic functions, myelination, plasticity and metabolic processes [17-19] and expresses a robust density in the hippocampus and cerebral cortex [20,21]. Compromised signaling of insulin receptors can result in cognitive deficits [22,23] and insulin signaling has been shown to regulate dopamine-mediated neurotransmission in animal models [24], influence the function of norepinephrine and serotonin transporters and consequently extracellular levels of norepinephrine and serotonin [25].

A key regulatory event of neuronal insulin receptor function, the insulin-stimulated autophosphorylation of the insulin receptor kinase at tyrosine residues, was lately found to be dose-dependently activated by dicholine salt of succinic acid (dicholine succinate, DS) suggesting the importance of DS in the mitochondrial biochemical pathway [26-28]. The presence of DS, and other respiratory substrates, stimulates insulin-dependent H_2_O_2_ production in the mitochondrial respiratory chain leading to an enhancement of insulin receptor autophosphorylation in cerebellar neurons [28,29]. As DS elevates the insulin-stimulated non-basal autophosphorylation of the insulin receptor it is considered an important endogenous sensitizer of the neuronal insulin receptor. Several studies demonstrate the biological effects of neuronal insulin receptor stimulation, via the mitochondrial respiratory chain using endogenous and exogenous compounds, and suggest that these effects are implicated in the stress response and the pathogenesis of a depressive-like state. For instance, thiazolidinediones, which act as potent sensitizers of the neuronal insulin receptor, enhance brain glucose utilization though increased neuronal mitochondrial biogenesis [30], decrease neuronal damage [31] and evoke anti-inflammatory effects [32-34]. Rosiglitazone, one of the insulin sensitizers of the thiazolidinedione class, has been found to induce an antidepressant-like effect in the tail suspension and forced swim tests in mice [35]. Importantly, this drug and another insulin receptor sensitizer, pioglitazone, were recently reported to be effective for the treatment of a major depressive disorder that was refractory to standard antidepressant treatment and accompanied by insulin resistance [36,37].

Intraperitoneal administration of DS for 7 days at doses of 10 and 25 mg/kg/day, but not at 1 mg/kg, rescued a 30%-decrease of brain N-acetylaspartate/creatine, a marker of neuronal function and viability, in middle-aged C57BL/6 N mice. In rats, the same treatment at all three doses rescued learning in step-through passive avoidance and a 4-day Morris water maze test; additionally, brain levels of N-acetylaspartate/creatine were also increased which were compromised in a model of chronic cerebral hypoperfusion [28]. Similarly, treatment with the highest dose of DS used in this study, resulted in a recovery of the acquisition of the step-through passive avoidance task and choline acetyltransferase activity which were suppressed in a rat model of beta-amyloid peptide-(25–35)-induced toxicity.

The current study's primary objectives were to evaluate the effects of intraperitoneal administration of DS for 7 days at a dose of 25 mg/kg/day on the development of depressive syndrome in a mouse chronic stress model and to relate this state to deficits in the step-down inhibitory avoidance learning model [38,39] in addition to anxiety-like behavior in the dark/light paradigm. A secondary objective was to investigate the hippocampal gene expression of insulin-like growth factor two (IGF2), a member of the receptor tyrosine kinase family, which is related to insulin signaling and similarly to the effects of DS on the mechanisms of cholinergic neurotransmission [40]. In a separate study, we observed that Illumina analysis pointed to enhanced hippocampal expression of this gene and related molecules of IGF1/IGF2 signalling following DS administration in chronically stressed mice with comparison to vehicle-treated animals (Strekalova and LePrince, unpublished results; Strekalova and Malin, *in preparation*). Importantly, IGF2 was found to interact with IGF1 and IGF2 receptor types in the brain to induce its biological effects [41]. IGF2 can effectively bind to the insulin receptor while alternative splicing revealed a difference in affinity for central and peripheral receptors and elucidated the structural determinants for high-affinity binding [42-44].

Here, we have used a variant of a recently validated mouse model of stress-induced anhedonia [45,46]. Anhedonia, a decreased ability to experience pleasures, is a core symptom of human depression [47], which in rodents is regarded to be reflected by a decreased intake of sucrose or other palatable solutions [48,49] and is reversible by antidepressants [5,50,51]. In the paradigm employed here, anhedonia, which is defined as a decrease below 65% in sucrose preference over water, occurs in a subgroup of animals. The anhedonic group, unlike the non-anhedonic group, exhibits increased floating in the forced swim test and disruptions in novelty exploration, contextual learning in the step-down inhibitory avoidance test and LTP in the CA1 area of the hippocampus. Furthermore, the anhedonic group also displays changes in EEG sleep patterns correlating to those seen in depressed humans. Chronic treatment with citalopram and imipramine counteracts the manifestation of stressed induced depressive-like traits in mice [45,52-54]. In this study, a 10-day stress protocol comprises of night time rat exposure and day time application of social defeat. Induction time of anhedonia was considerably shortened by the dual application of stressors, each of which was shown to effectively induce a hedonic deficit [15,46]. Intraperitoneal administration of DS at 25 mg/kg/day for 7 days was chosen since the same treatment was found to induce neurochemical alterations and beneficial behavioral effects lasting up to at least two weeks [28]. As a reference, the tricyclic imipramine (7 mg/kg/day) was delivered via drinking water 1 week before the onset of stress and throughout the entire stress procedure in accordance to a previously validated protocol [53]. Separate studies using the same model as the current study showed that one-week pre-treatment with daily intraperitoneal imipramine injections (15 mg/kg/day) in CD1 mice significantly attenuated stress-induced changes in the sucrose and forced swim test as compared with vehicle-injected animals [53].

## Methods

### Animals and housing

For the chronic stress experiment, we used male CD1 mice, widely used in behavioral, biochemical and molecular research. Male mice (age: 3 months) were purchased from Charles River (Sulzfeld, Germany). Ten days before the behavioral experiments, mice were housed single-caged under a reverse 12 h : 12 h light–dark cycle (lights on: 21:00 h) in standard laboratory conditions (22 ± 1°C, 55% humidity, food and water ad libitum). All experiments were carried out in accordance with the European Committees Council Directives and had been approved by the Animal Experimental Committee of Claude Bernard University of Lyon and the Animal Ethical Committee of the University of Maastricht.

### General conditions of experiment

**Figure 2:**
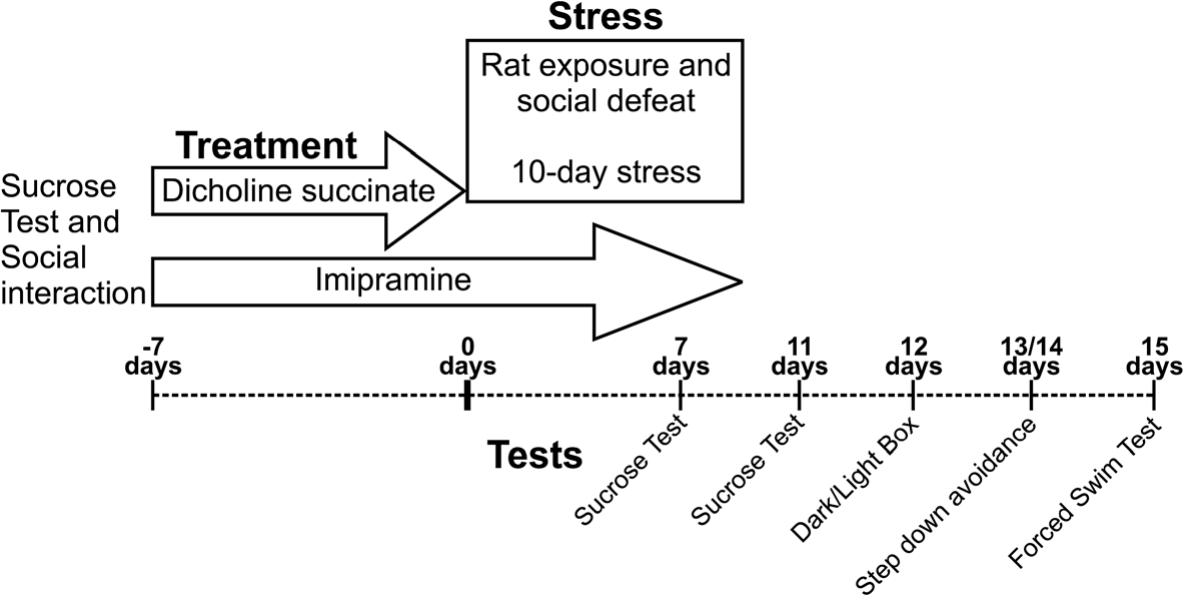
**Timeline of study.** Application of stress, drugs and behavioral testing in the chronic stress study.

Parameters of social behavior were determined one week before the chronic stress procedure in a social interaction test as described elsewhere [46,53]. Body weight and baseline preference to a 1% sucrose solution (*see* Sucrose Test) were evaluated as well. The experimental and control groups were balanced upon these parameters [46,53]. Together, 75 mice were assigned to a stress group and 25 controls constituted a non-stressed control group. Among animals from a stress group, twenty five mice received either no treatment, were treated with imipramine or with DS. Control mice were either not treated (n = 8), treated with imipramine (n = 8) or DS (n = 9). In control and stress groups, imipramine (7 mg/kg/day) was administrated via drinking water starting 7 days prior the onset of stress and lasting the entire duration of the stress procedure (Figure 2).

The current reference antidepressant treatment was selected because of its maximal effects in lowering the rate of stress-induced anhedonia over other methods of delivery and doses of antidepressants [53,55]. Previous experiments revealed a weaker effect of one-week antidepressant pre-treatment with daily i.p. injections of imipramine (15 mg/kg/day) in CD1 mice for stress-induced depressive-like changes [53]. DS was administrated during 7 consecutive days preceding chronic stress. Additionally to baseline measurements, sucrose consumption tests were performed after 7 and 10 days of the chronic stress procedure (*see Rat exposure while in a small container, Social defeat stress*); animals were weighed weekly during the study and at the end of the stress protocol. On day 2 after stress, animals were tested in the dark/light test; on days 3 and 4, step down avoidance test was performed. Five days after the termination of the stress procedure, mice were tested in the forced swim test (*see below*) and on the next day sacrificed for gene expression analysis (*see below*; Figure 2).

### Chronic stress experiment

The chronic stress procedure lasted 10 days (Figure 2). Each day, stressors were used in the same sequence: between 18.00 and 9.00 rat exposure while in a small container was applied. 30-min sessions of social defeat were employed twice daily between 11:00 and 16:00. An inter-session interval between the application of two social defeat stress sessions and between social defeat and rat-exposure procedures was at least 2 h.

### Rat exposure while in a small container

Mice were introduced to transparent glass cylindrical containers (15 cm × Ø 8 cm) and placed into the rat cage (15-h exposures were performed between 18.00 - 9.00).

### Social defeat stress

Social defeat procedures took place during the dark phase; to enable a visual control over the resident-intruder confrontation, the test was carried out under red light. In a preliminary test, aggressive individuals of the CD1 mouse strain that were able to attack the counter-partners in less than 60 s without injuring them were selected for this procedure similar to commonly used protocols of social defeat stress [56,57]. These animals were introduced in the home cages of mice from the stress group during social defeat sessions, deviating from originally proposed paradigms of social defeat [58] but broadly used in a variety of experimental situations [57,59,60]. Social interaction was set up in the home cage of stressed animals as it enhances the impact of the stress procedure in a lasting manner. In a variant procedure, a defeated animal is left in chronic contact with the olfactory cues of the aggressive intruder, such that exposure to a psychological stressor is chronic although the actual agonistic experience is intermittent. Average duration of each session was 30 min in accordance with commonly used protocols [58,60]. During social defeat stress, test mice typically showed flight response, submissive posture and vocalization. Pairs of animals were carefully observed in order to exclude any physical harm. In rare cases of its incidence, aggressive individuals were immediately removed from the cage of resident mice. Total duration of social defeat stress was 10 days, congruent with previously published studies that were using aggressive CD1 mice [53,55].

### Sucrose test

Mice from all groups were simultaneously given for 8 h (between 9.00 – 17.00 h) a free choice between two bottles, one with 1%-sucrose solution and another with tap water*.* To prevent possible effects of side-preference in drinking behavior, bottle position was switched after 4 h. No previous food or water deprivation was applied before the test. To minimize the spillage of liquids during sucrose test, bottles were filled in advance and kept inverted for at least 12 h prior to testing in the same room where testing took place. This method was shown to ensure a low error of measurement (up to 0.1 ml). To decrease the variability in sucrose consumption during the very first exposure to sucrose solution (baseline sucrose test), 18 h before baseline animals were allowed to drink a 2.5% sucrose solution in a one-bottle paradigm for 2 h. The intake of water and sucrose solution was estimated by weighing the bottles before and after free access to the liquids. Sucrose preference was calculated as a percentage of the consumed sucrose solution from the total amount of liquid drunk:

$$\begin{array}{ll} \text{Sucrose Preference} & =\left[\text{V},\left(\text{Sucrose solution}\right),/\right)\\ & \;\left(\text{V},\left(\text{Sucrose solution}\right),+,\text{V},\left(\text{Water}\right)\right]\\ & \;\times 100\% \end{array}$$

A decrease of sucrose preference to a level below 65% measured at the 10^th^ day of continuous stress application was taken as a criterion for anhedonia. This criterion was based on the fact that none of the control animals exhibited < 65% preference for sucrose at that time point of the study. In addition, our previous results indicated that mice matching this criterion showed a depressive-like syndrome [53,61].

### Forced swim test

Forced swim test was performed five days following termination of the stress procedure as previously described [46,53]. We used a large size pool (square pool: 21 cm × 42 cm × 15 cm) illuminated with red lighting, water temperature was kept at 30°C and water height was 10 cm. The modified forced swim test employed here was shown to prevent behavioral artifacts in this test caused by chronic stress-induced hyperlocomotion. Previous studies show that, with standard protocols of the forced swim test [62], chronically stressed mice exhibit hyperactivity which masks depressive-like behaviors in most behavioral tests [62]. For instance, chronically stressed C57BL6N mice show increased swimming behavior in brightly illuminated pools that is abolished with a low dosage of diazepam or testing these animals under red light [62]. Mice were introduced 2 min to the pool for a single swimming session. The latency of the first episode of floating determined as absence of any directed movements of the animals heads and bodies (duration more than 3 sec) and duration of floating were scored off-line. Visual scoring was validated as described elsewhere [63] with CleverSys software (CleverSys, VA, USA).

### Dark–light box

The dark/light box consisted of two Plexiglas compartments, one black/dark (15 cm × 20 cm × 25 cm) and one lit (30 cm × 20 cm × 25 cm) connected by a tunnel. Mice were placed into the black compartment from where they could visit the lit box illuminated by a light intensity of 5 Lux. Our studies showed that with the classical protocol of the dark–light box, chronically stressed mice exhibit light-induced hyperlocomotion that confounds the evaluation of anxiety-related behaviors in this paradigm [62]. In chronically stressed C57BL6N mice, increase in time spent in the lit compartment under lighting of 600 Lux is abolished with a low dosage of diazepam; the same effect as the treatment can be achieved by dimming down the illumination or using red light [62]. Latency of the first visit to the lit box, total duration spent therein and number of visits to this anxiety-related area were scored by visual observation over 5 min.

### Step-down inhibitory avoidance learning test

Control, stressed non-anhedonic and stressed anhedonic mice were analyzed for hippocampus-dependent memory in a step-down inhibitory avoidance paradigm. The step-down apparatus (Technosmart, Rome, Italy) consisted of a transparent plastic cubicle (25 cm × 25 cm × 50 cm) with a stainless-steel grid floor (33 rods 2 mm in diameter) onto which a square wooden platform (7 cm × 7 cm × 1.5 cm) was placed. A shocker was used to deliver an alternating electric current (AC, 50 Hz, Evolocus, Terrytown, NY, USA). In this paradigm, animals were trained not to step down from a platform onto a grid floor to avoid an electric shock. During the training session, mice were placed onto the platform inside a transparent cylinder for 30 s to prevent them from immediately stepping down. After removal of the cylinder, the time until the animal left the platform with all four paws was measured as baseline latency of step down. Immediately after step down, mice received a single electric foot-shock (0.5 mA, 2 sec) and returned to their home cages. Twenty four hours later, during the recall trial session, animals faced the same context as in the training session. Latency of step down with all four paws was measured until 180 s elapsed. According to previously validated criteria for the acquisition of the step down avoidance task [38,39], an increase of latencies measured in animals during a recall session are taken as a sign of long-term learning.

### Administration of compounds in chronic stress study

Imipramine (Sigma-Aldrich, St. Louis, MO, US) was dissolved in tap water; the solution was freshly prepared every 2–3 days. Since imipramine is light sensitive, bottles were protected by aluminum covers. The calculation of the concentration of imipramine in drinking water was based on the previously evaluated mean volume of daily water consumption in CD1 mice that was about 3.5 ml and on the dosage of treatment. Dosage for imipramine was set at 7 mg/kg/day as previous studies showed that chronic administration of imipramine at15 mg/kg/day with drinking water, but not 7 mg/kg/day, significantly affects sucrose intake and locomotor behaviour in naïve C57BL/6 N mice [55]. Imipramine was delivered with drinking water starting 1 week before the onset of stress and then throughout the entire duration of the chronic stress procedure. DS, provided by Buddha Biopharma Oy Ltd (Helsinki, Finland), was dissolved in water for injection and administrated via daily i.p. injections at 25 mg/kg/day for 7 consecutive days; this scheme of treatment was demonstrated to evoke memory-enhancing effects in mice and rats and neurochemical effects lasting over a period of two weeks [28]. The volume of DS and vehicle injections was 0.01 ml/g body weight 0.01 ml/kg.

### Brain dissection

Mice were sacrificed by cervical dislocation. The brains were quickly removed and dissected on ice, dissected hippocampi material were kept frozen at −80°C.

### Real-time PCR assay

Total RNA was isolated from mouse brain using RNeasy RNA extraction kit with DNaseI treatment following the manufacturer's instructions (Qiagen, Hilden, Germany). Using random primers and Superscript III transcriptase (Invitrogen, Darmstadt, Germany), 1 μg total RNA was converted into cDNA. Specific primers for IGF2 gagttcagagaggccaaacg (forward), ttagtgtgggacgtgatgga (reverse) were purchased from Sigma-Aldrich (Sigma-Aldrich, St. Louis, MO, US). The housekeeping gene glyceraldehydes-3-phosphate dehydrogenase (GAPDH) was used as a reference gene for quantification. PCR was performed with 50 ng cDNA in a 25 μl reaction volume containing a SYBR Green Master Mix (Roche, Mannheim, Germany). Amplification was carried out utilizing a Roche LightCycler 480 sequence detection system (Roche). Cycling conditions were 50°C for 2 min, 95°C for 10 min followed by a 40-cycle amplification at 95°C for 15 s, and 57°C for 1 min. Experiments were repeated two times and samples were analyzed in triplicate. Results of the real-time PCR data were represented as Ct values, where Ct is defined as the threshold cycle of PCR at which amplified product was first detected. To compare the different RNA samples, we used the comparative Ct method and compared the RNA expression in samples to that of the control in each experiment.

### Statistical analysis

Data were analyzed with GraphPad Prism version 5.00 for Windows (San Diego, CA, USA) using two-way ANOVA followed by Bonferroni post-tests; where appropriate, one-way ANOVA with Tukey's Multiple Comparison Test was utilized while Fisher's exact test was used to compare group size. The level of confidence was set at 95% (p < 0.05) and data are shown as mean ± SEM unless otherwise stated.

## Results

### Assessment of anhedonia induction

**Figure 3:**
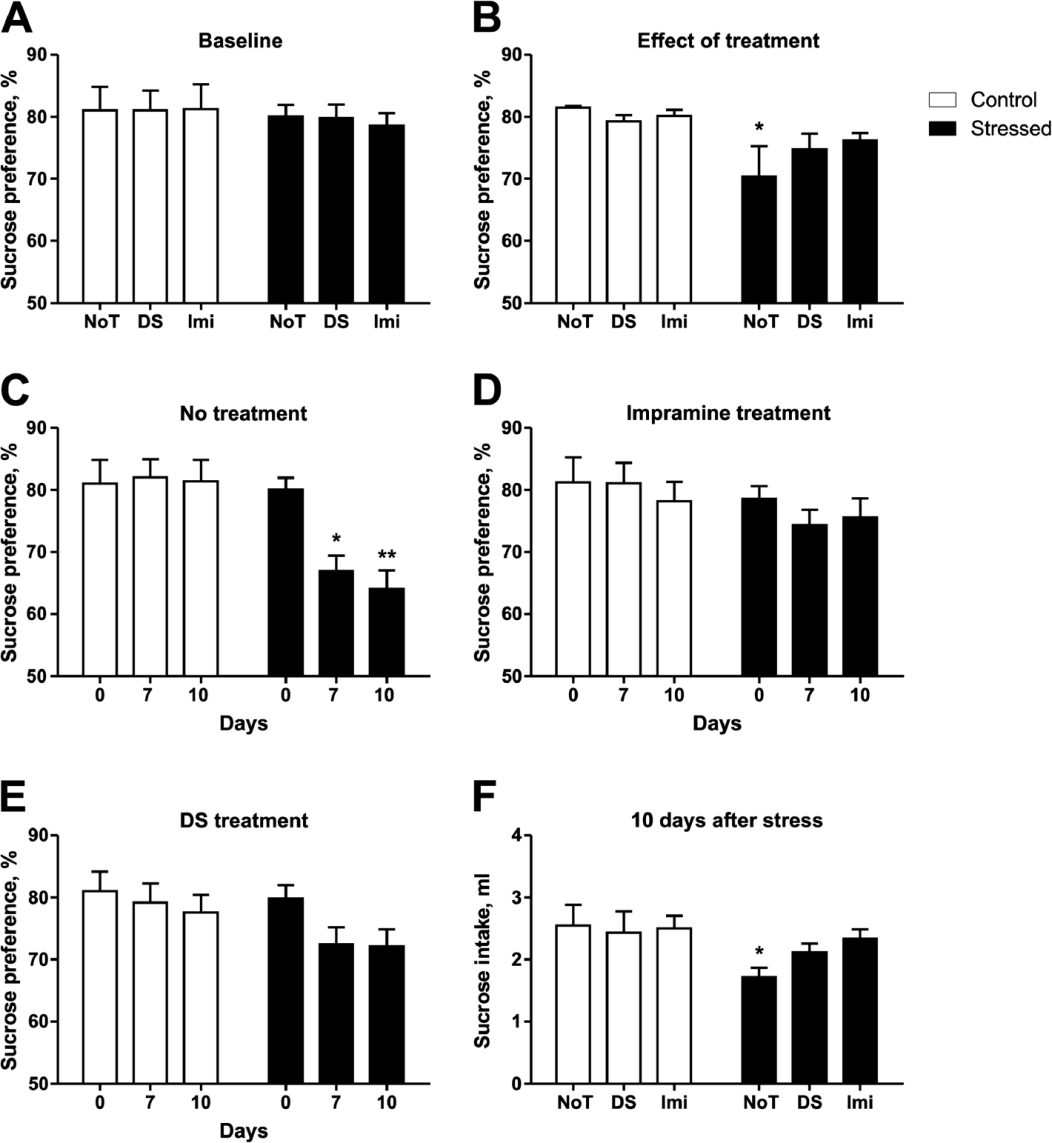
**Imipramine and dicholine succinate counteract stress-induced decrease in sucrose preference and sucrose intake.** (**A**) Groups of mice assigned for planned treatment had similar means of sucrose preference before the beginning of dosing. (**B**) Two-way ANOVA revealed a significant effect for the stress condition in sucrose preference *F* = 11.9, *DFn* = 1, *DFd* = 12, *p* =0.0058 ^*^ vs respected control. (**C**) NoT stress mice showed a significant reduction for sucrose preference at days 7 and 10 in relation to the stress condition *F* = 12.75, *DFn* = 1, *DFd* = 87, *p* =0.0006, Bonferroni day 7, *p* < 0.05 ^*^ respected control; day 10, *p* < 0.01 ^**^ vs control. (**D**,**E**) Imi and DS stress mice did not show significant differences neither at day 7 nor day 10 for the stress condition *F* = 1.686, *DFn* = 1, *DFd* = 89, *p* = 0.1975 and *F* = 2.252, *DFn* = 1, *DFd* = 90, *p* = 0.1369 respectively. (**F**) Following chronic stress, the total sucrose intake was measured and two-way ANOVA revealed an overall condition effect while Bonferroni showed a significant reduction in sucrose intake only in the NoT stress group *F* = 5.352, *DFn* = 1, *DFd* = 88, *p* = 0.0230, Bonferroni: NoT *p* < 0.05, DS, Imi *p* > 0.05 ^*^ vs respected control. NoT: non-treated group; Imi: imipramine-treated group, DS: DS-treated group. Data is shown as mean ± SEM.

Initially, mice assigned to distinct experimental groups had a similar sucrose preference (Figure 3A), intake of sucrose solution and water, as well as body weight (*data not shown*). Two-way ANOVA revealed a significant difference between stress and control groups (*F* = 11.9, *DFn* = 1; *DFd* = 12, *p* =0.0058) while Bonferroni's indicated that the non-treated stressed group had a significant decrease in sucrose preference (*p* < 0.05) unlike the imipramine and dicholine succinate treated groups which were not significant; indicating the ability of DS, like imipramine, to prevent a reduction in sucrose preference for the stressed cohort (Figure 3B). During the chronic stress study, sucrose preference was assessed at day 7 and 10 (end of stress, Figure 3C, D E) affirming that only the non-treated stress group showed a significant decrease in sucrose consumption (*F* = 12.75, *DFn* = 1, *DFd* = 87, *p* = 0.0006) and at day 10 the mean for the non-treated stress group (mean = 64.06) dropped below the defined threshold for anhedonia (sucrose preference below 65%). Neither the imipramine nor the DS treated stressed groups showed a significant change in sucrose preference at either day (*F* = 1.686, *DFn* = 1, *DFd* =89, *p* = 0.1975 and *F* = 2.252, *DFn* = 1, DFd = 90, *p* = 0.1369, respectively) reiterating the ability of both DS and imipramine to prevent a decrease in sucrose preference for the stressed animals.

Previous studies have evidenced the significance of absolute sucrose intake as a parameter sensitive to the effects of antidepressants in chronic stress paradigms [53,64]. A counteraction of stress-induced decrease in absolute sucrose intake is taken as a sign of antidepressant-like effects of the treatment [48]. Following the stress procedure, absolute sucrose intake was measured (Figure 3F) and two-way ANOVA with Bonferroni's post-test attested that only non-treated stress mice had a significant decrease in sucrose intake (F = 5.352, DFn = 1; DFd = 88, p = 0.0230, Bonferroni: NoT p < 0.05, DS, Imi p > 0.05) The total number of anhedonic mice observed among the imipramine-treated (n = 6) and DS-treated (n = 8) stressed groups was lower than in the non-treated stressed group (n = 14, p = 0.02 and p = 0.08, respectively, Fisher's exact test) further suggesting that both compounds counteract a development of stress-induced anhedonia.

### Effects of treatment on floating behavior

**Figure 4:**
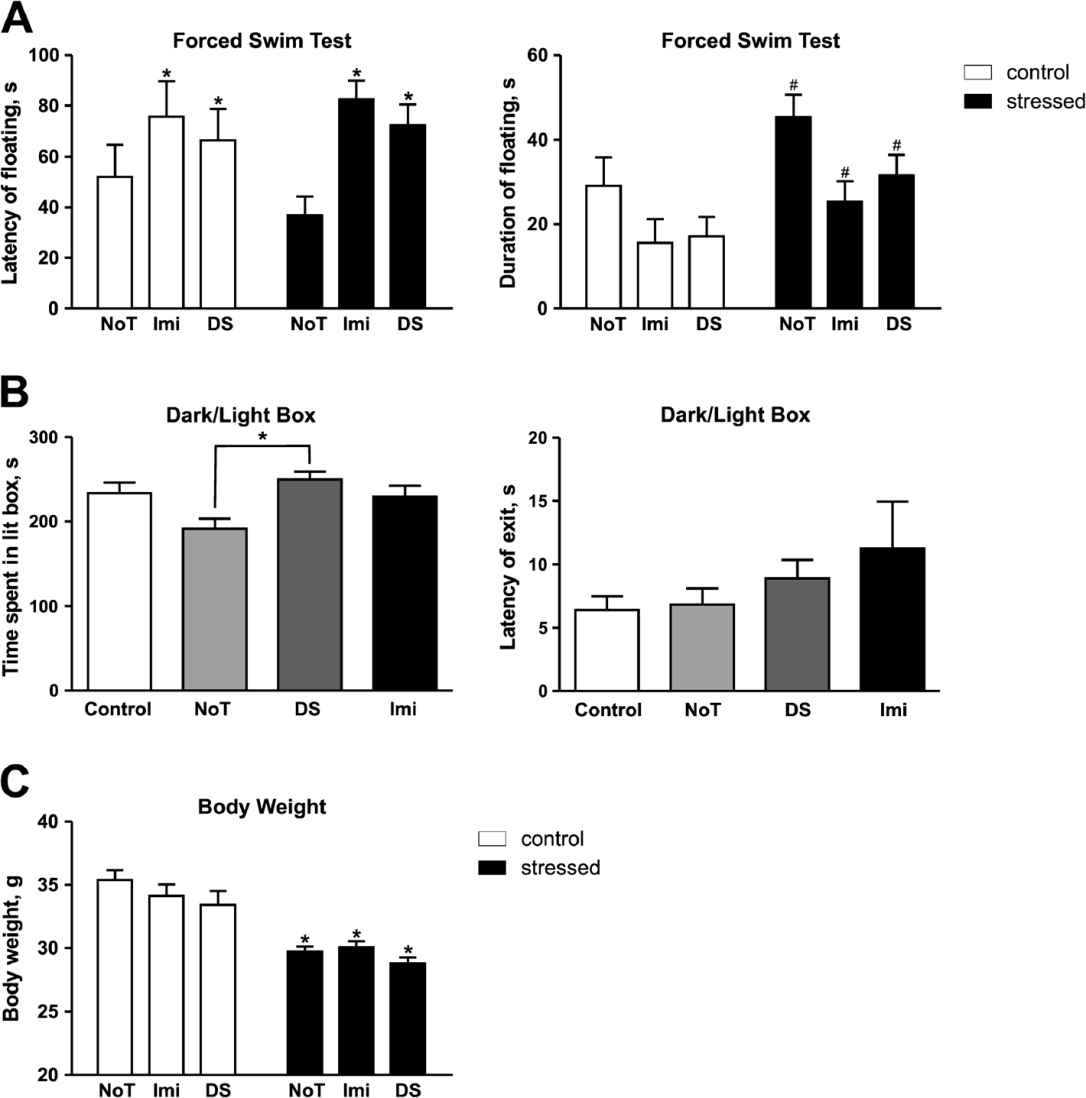
**Dicholine succinate reduces stress-induced floating and anxiety-like behaviors, but not a decrease of body weight.** (**A**) Latency of floating had an overall significant difference for treatment while the duration of floating had an overall significant difference for the stress condition *F* = 4.652, *DFn* = 2, *DFd* = 89, *p* = 0.0120 and *F* = 5.333, *DFn* = 1, *DFd* = 89, *p* = 0.0232 respectively two-way ANOVA; * vs non-treated, # vs respected controls. (**B**) ANOVA revealed a significant difference for time spent in the lit compartment with Tukey showing only a difference between the Not and DS treated stress groups *F* = 4.469, *DFn* = 3, *DFd* = 39, *p* = 0.0086, Tukey *p* < 0.01 ;*vs respected control. No significant difference in latency of exit was observed. (**C**) Two-way ANOVA revealed a significant difference in body weight for the stress condition in all groups *F* = 66.81, *DFn* = 1, *DFd* = 89, *p* < 0.0001. ^*^ vs respected controls NoT: non-treated group; Imi: imipramine-treated group, DS: DS-treated group. All data is shown as mean ± SEM.

Latency to floating was significantly altered by treatment in the forced swim test as revealed by two-way ANOVA (*F* = 4.652, *DFn* = 2, *DFd* = 89, *p* = 0.0120); however, Bonferroni's post-test did not detect any differences between stressed and control groups suggesting that non-treated animals were significantly faster to quit swimming compared with treated animals. As with latency to floating, the duration of floating was significantly divergent between control and stress cohorts but no differences were seen between groups (*F* = 5.333, *DFn* = 1, *DFd* = 89, *p* = 0.0232, Figure 4A). The effect of treatment was robust albeit not significant (*p* =*0.0590*) indicating that the stressed condition had a significant effect on floating duration while non-treated animals had a very strong tendency for increased periods of floating suggesting that DS and imipramine treatment both had a positive antidepressant-like effect.

### Evaluation of anxiety scores

The level of anxiety was significantly altered between groups as revealed by total duration spent in the lit compartment (one-way ANOVA, *F* = 4.469, *DFn* = 3, *DFd* = 39, *p* = 0.0086, Figure 4B) Tukey's post-test showed that the non-treated stress group spent a very significantly diminished duration (*p* < 0.01) in the lit compartment compared with the DS treated stress group; no significant differences were revealed amidst the other groups indicating the ability of DS to block stress-induced anxiety. Latency to exit was not different between any of the groups (one-way ANOVA, *F* = 1.232, *DFn* = 3, *DFd* = 41, *p* = 0.3103).

### Changes in body weight

After the termination of stress, body weight was significantly shifted between control and stress animals and all stress groups showed an extremely significant decrease in body weight compared with controls (two-way ANOVA, *F* = 66.81, *DFn* = 1, *DFd* = 89, p <0.0001, Bonferroni, NoT, Imi, DS *p* < 0.001, Figure 4C).

### Step-down avoidance learning

**Figure 5:**
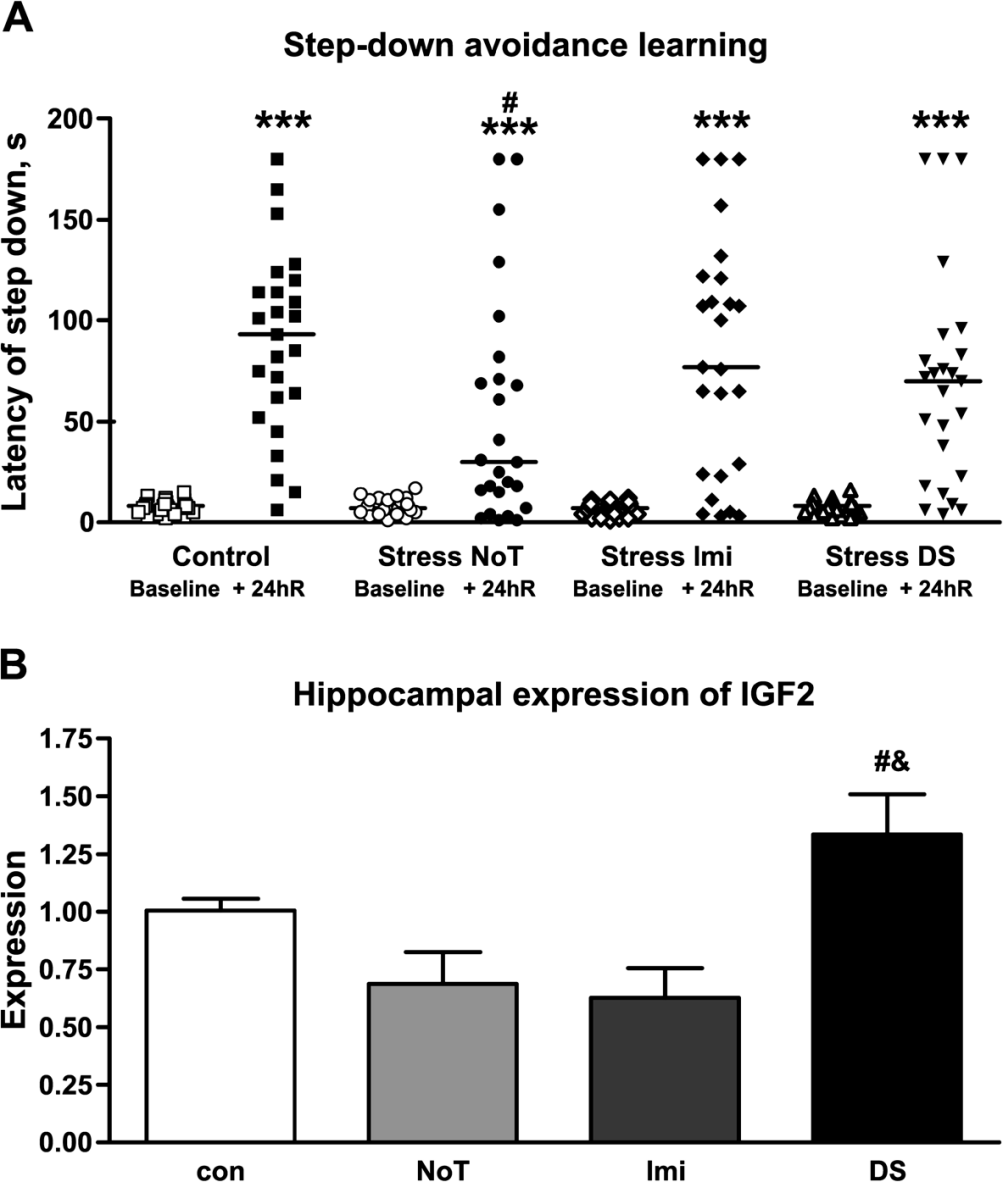
**Effect of imipramine and dicholine succinate on step-down avoidance learning and hippocampal expression of IGF2.** (**A**) All groups showed a significant increase in the latency to step down as compared to their respective baselines one-way ANOVA *F* = 23.27, *DFn* = 7, *DFd* = 192, *p* < 0.0001 *** vs baseline while the NoT group was significantly lower for the recall test compared to control indicating a disruption in contextual learning # vs control. (**B**) There was an overall significant difference seen in the hippocampal gene expression for IGF2, while Tukey's post-test revealed differences for IGF2 gene expression between DS and Imi treated groups and DS and NoT treated groups p < 0.01, *F* = 6.232, *DFn* = 3, *DFd* = 33, *p* = 0.0018. # vs NoT & vs Imi; NoT: non-treated group; Imi: imipramine-treated group, DS: DS-treated group. All data is shown as mean ± SEM.

All stress groups tested showed a significant difference on test day as compared to the baseline day (one-way ANOVA, *F* = 23.27, *DFn* = 7, *DFd* = 192, *p* < 0.0001, Figure 5A) suggesting that all groups were able to acquire the task while no observed difference in baseline shows that initial anxiety levels were the same between groups. Tukey's post-test revealed that non-treated stress animals were significantly different compared to controls in the 24 hr recall showing reduced latencies to step down (*p* < 0.05) whilst imipramine and DS treated stress animals did not show any difference, thus suggesting stress disrupted learning in non-treated animals and demonstrating the ability of DS and imipramine to preserve contextual memory for stressed animals.

### Hippocampal gene expression of insulin-like growth factor 2

IGF2 expression levels were measured following the stress procedure. Hippocampal expression levels of IGF2 were significantly altered in stressed animals (one-way ANOVA, *F* = 6.232, *DFn* = 3, *DFd* = 33, *p* = 0.0018 Figure 5B). Post-test with Tukey revealed that DS treated animals had a significant increase in IGF2 expression levels compared with non-treated and imipramine treated groups (*p* < 0.01) while no significant differences were observed between other groups.

Altogether, pre-stress treatment with imipramine and DS prevented a stress-induced decrease of sucrose intake and preference, counteracted a stress-induced increase in floating, conserved contextual inhibitory learning and averted anxiety-like behavior; however, the applied drugs did not prevent a loss in body weight. A pronounced up-regulation of IGF2 gene expression in the hippocampus accompanied these behavioral effects of DS treatment in chronically stressed mice.

## Discussion

Initial values of sucrose test parameters were similar between all groups (Figure 3A) and stress exposure lowered sucrose preference in agreement with other reports [49,61,65]. Stressed mice treated with DS showed no significant change in sucrose preference measured on the 7^th^ and 10^th^ day of stress as compared to control animals (Figure 3E). Administration of the classical antidepressant imipramine resulted in a similar effect. Earlier, we have shown in a model of stress-induced anhedonia that the stress-induced decrease in sucrose preference is paralleled by a reduction in sucrose intake [46,53]. In particular, a disruption of synaptic plasticity and pronounced behavioral despair in the forced swim test were observed in mice, which showed a decrease in both sucrose intake and preference but not a reduced sucrose preference alone, which occurs exclusively at the expense of high water intake [45,51,53]. Thus, the partial preclusion of the stress-induced reduction for both sucrose preference and consumption by treatment with imipramine and DS manifests their antidepressant-like activity in our study. Importantly, administration of imipramine and DS did not alter sucrose test parameters in control animals ruling out any possible confounding artifacts for sucrose test measurements which could be related to treatment. Imipramine, used as a reference drug in this experiment, is well documented to counteract the stress-induced decrease in sucrose intake and preference seen in rodents [51,65]. Overall, our data suggest that chronic administration of imipramine and DS has the potential to counteract the development of stress-induced anhedonia, i.e. elicits an antidepressant-like activity in the mouse paradigm employed in the present study.

Described above evidences, for antidepressant-like effects of DS in the sucrose test, are in line with the outcome from the forced swim test. While the effects of chronic stress on the latency to the first episode of floating were not significant, DS and imipramine treated mice had significantly higher values of this parameter (Figure 4A). DS administration strongly reduced the duration of floating in the chronic stress experiment suggesting that treatment with DS prevents a stress-induced state of behavioral despair as measured by elevated floating behavior (Figure 4A) [45,46,48,66]. In the current work, these antidepressant like effects observed with DS administration were also demonstrated for treatment with imipramine; earlier, we reported congruent changes in the forced swim test following chronic administration of citalopram [53]. Coinciding with these results, another insulin sensitizer, rosiglitazone, was reported to reduce immobilization and floating behaviors in mouse tail suspension and forced swim tests respectively [35].

Both treatment with imipramine and DS decreased anxiety scores as shown by increased time spent in the lit compartment in the dark/light box indicating their anxiolytic and anti-stress effects (Figure 4B). Such effects are well documented for imipramine and other tricyclics [48]. Elevated anxiety was found to parallel anhedonia induction in chronic stress models [15,45,48,49].

In the present study, the stress-induced loss in body weight does not correlate with an occurrence of anhedonia and depressive-like syndrome in mice. However, it has been shown that antidepressant effects of pharmacological treatment parallel a restoration of body weight [48,67]. We did not find such an effect with imipramine and DS treatment (Figure 4C) in the present study but it must be noted that a lack of positive effects on the restoration of body mass in depressed patients is well documented for many antidepressants including fluoxetine and other SSRIs [48,66].

Treatment with imipramine and DS prevented stress-induced memory impairment in the step-down inhibitory avoidance task (Figure 5A). The latency of step down measured twenty four hours after training session was previously validated as a reliable measure of hippocampus-dependent performance in mice [38,39]. Deficits in hippocampus-dependent performance were earlier shown to be a specific feature of stressed anhedonic mice as compared to stressed individuals without a depressive syndrome in various chronic stress paradigms. Treatment with citalopram was shown to rescue contextual fear conditioning in a model of stress-induced anhedonia [38,45,53]. Since imipramine administration also precluded deficits in the step-down avoidance test, beneficial effects of DS on learning can be accounted for by its antidepressant action which is generally accompanied by an improvement in cognitive function in clinical and pre-clinical studies. These data are in line with ameliorative effects of DS on hippocampus- and cortex-dependent learning in step-though and Morris water maze paradigms which this drug exerted under pathological conditions of diverse origins [28,29].

Our study evidenced a sharp increase in hippocampal gene expression of IGF2, a member of the insulin gene family with known neurotrophic properties, after administration of DS, but not imipramine, in stressed mice (Figure 5B). Utilizing the hippocampi of mice from the same experimental groups (five animals per each group were analyzed), gene expression profiling was performed using Illumina technology (Integragen, Evry, France and Northwestern Chicago University, USA). This study revealed significant effects of DS on the expression of a number of functionally important genes (Strekalova and Malin, *in preparation*). Therefore, total RNA was isolated using RNeasy Mini Kit (Qiagen, Hilden, Germany). Total RNA samples were hybridized to IlluminaBeadChips (MouseRef-8 v2 Expression BeadChip; Illumina, Inc. San Diego, CA, USA) which were prepared using the IlluminaTotalPrep RNA Amplification kit (Applied Biosystems/Ambion, Carlsbad, CA, USA); the samples were assigned to the chips in random order with the constraint that no two samples from the same group were assigned to the same chip, to avoid confounding of experimental groups with the chips. Microarray data were analyzed using standard analysis procedures which included assessment of the overall quality of array data and statistical evaluation of differentially expressed genes (Integragen, Evry, France). Once the quality of array data was confirmed, the Gene Chip Operating System (Illumina, Inc. San Diego, CA, USA) was used to calculate signal intensities, detection calls, and their associated P values for each transcript on the array. Gene expression was normalized to the expression of the house gene beta-actin, due to its stable expression, and calculated as percent mean of the control group. Differences in gene expression between groups were evaluated using ANOVA followed by Fisher's Least Significant Difference test.

In line with the outcome from mRNA evaluation (Figure 5B), these data revealed a significant increase of IGF2 expression in stressed DS-treated mice (163.1 ± 30.17% from control) and its significant decrease in stressed mice that were not treated (83.0 ± 4.49% from control). This study indicated that stressed DS-treated mice showed a significant expression enhancement of Htra1, HtrA serine peptidase 1 which cleaves IGF-binding proteins (IGFBPs) from IGF1 and IGF2 and activates these factors, in comparison to non-treated mice (144.3 ± 9.16 vs. 119.2 ± 17.6% from control, respectively). Preliminary data showed no such changes of IGF2 in the non-stressed control group. Also, this experiment revealed significant effects of DS on the expression of other elements of the IGF1/IGF2 system in chronically stressed mice, including Htra1, as well as IGF1 and IGF1 receptor, the insulin receptor and several insulin-like growth factor binding proteins. Again, no such changes were detected in non-stressed mice treated with DS. While it is important to study above-mentioned findings with additional methods, they generally support our data on elevated mRNA of IGF2 in the hippocampus of DS-treated stressed mice and suggest this elevation to be a part of systemic changes in IGF1/IGF2 signaling in these animals. A lack of such molecular effects in naïve mice treated with DS might be due to distinct functional states of the IGF1/IGF2 system during stress and resting conditions; whereas, the activation of this signaling might occur as an adaptive mechanism in response to biological challenges.

A comparison of pharmacologically naïve anhedonic versus resilient animals in changes of the above-mentioned elements of IGF1/IGF2 signaling speaks in favor of the latter view. Our studies revealed an intriguing difference in the IGF2 expression between non-treated stressed anhedonic and resilient animals (58.8 ± 2.32% vs. 107.2 ± 8.7% from control, respectively), suggesting the elevated IGF2 to be a correlate of stress resilience while its decrease as a parallel of susceptibility to a depressive-like state. Moreover, the expression of Htra1 was reduced in non-treated anhedonic and essentially increased in the resilient group (91.08 ± 6.16% vs. 148.34 ± 11.16% from control, respectively). Anhedonic and non-anhedonic groups had differential expressions in most of the other above-listed elements of the IGF1/IGF2 signaling system (Strekalova and Malin, *in preparation)*. Thus, the outcome from the gene expression profiling experiment is in line with a suggestion that enhanced expression of IGF2 can mediate resilience to stress-induced anhedonia induced by administration of DS in our study.

IGF2 is widely expressed throughout the brain and is abundant in the hippocampus [68]. Various challenges such as acute hypoxia, exposure to toxicity stress and cerebral ischemia were shown to induce long-lasting changes in IGF2 expression which is considered to have an important neuroprotective function [69-71]. IGF2 was recently shown to be an important regulator of hipppocampal neurogenesis in the context of extinction in fear conditioning learning [72]. Moreover, IGF2 was shown to enhance adult neurogenesis [73]. Our results evidenced suppressive effects of stress on hippocampal levels of IGF2 demonstrating that chronic stress in mice has a tendency to decrease the content of this neurotrophic factor that might be associated with its above-mentioned role in the regulation of neurogenesis which is inhibited by stress [74]. In order to immediately address how crucial the role of IGF2 in the development of stress-induced depressive syndrome might be, a chronic intrahippocampal administration of this molecule could be applied with our model. Treatment with DS significantly elevated levels of IGF2 in stressed mice above that of controls. The mechanisms of this effect can be due to an earlier demonstrated DS-induced enhancement of choline content and acetylcholine function in the brain [28] since a functional link between this neurotransmitter system and IGF2 is particularly evidenced by increased expression of this neurotrophic factor after choline administration in the hippocampus and frontal cortex [40,75,76]. In these studies, choline supplementation increased levels of IGF2 in the hippocampus and changed expression of its receptors in the septum, it also enhanced IGF2-induced acetylcholine release and cholinergic neurontransmission [40]. Importantly, there was an increase of choline acetyltransferase activity after DS treatment in rats subjected to a toxic treatment with beta-amyloid peptide-(25–35) [28] and elevated IGF2 content in our study on mice were observed two weeks after the termination of a chronic DS administration (Figure 2).

Interestingly, recent results evidenced a critical role of IGF2 in inhibitory avoidance learning as shown in the fear conditioning paradigm [72,77] which can additionally explain the beneficial effects of DS on performance in chronically stressed mice in the step-down inhibitory avoidance task (Figure 5A). Taking these data into account and given the fact that hippocampal IGF2 signaling regulates adult neurogenesis in the context of fear extinction learning [73] it would be of high interest to assess the expression of IGF2 in the step-down avoidance inhibitory task that is similar to the fear conditioning paradigm form of contextual learning. However, the fact that imipramine did not evoke any effect on IGF2 gene expression while exhibiting both prominent anti-depressant and memory-enhancing effects similar to DS suggest that elevated expression of this molecule cannot be the sole mechanism of antidepressant and memory-enhancing effects observed for DS in the current study.

## Conclusions

Together, our data suggest that the chronic administration of DS in mice before the onset of stress exerts antidepressant-, anxiolytic-like and memory-preserving effects in a mouse chronic stress depression model similar to the classical antidepressant imipramine. The effects of DS parallel a lasting increase of hippocampal expression of IGF2 that is not observed in imipramine-treated mice suggesting distinct mechanisms of beneficial action of the two drugs used here in this model of experimental depression. The latest clinical study showed the efficacy of insulin receptor sensitizers in patients who were refractory to a standard antidepressant treatment; thus, arguing for a relevance of heterogeneity in neurochemical factors underlying this disorder and its cure [36,37].

To date, one can only speculate about the specific mechanisms of the reported effects of DS here. One can hypothesize that they are mediated by neurotrophins like IGF2 that activate neurogenesis and evoke anti-inflammatory effects [74,78,79] as well as associated with IGF2-induced changes in expression of GluR1 [77], GABA release and receptor expression [75,80] and activity of PKC- and calmodulin kinase I-dependent pathways [75,81]. Additionally, preliminary data suggest anti-inflammatory effects of DS and other insulin receptor sensitizers which were used in clinical [32,34,37] and pre-clinical studies [82]; this can per se, underlie an antidepressant effect taking into account emerging evidence for the role of inflammatory mechanisms in depression [83,84].

Meanwhile, alterations in insulin signaling pathways by DS may also represent an important part of the underlying antidepressant action. A recently found antidepressant effect of the neuronal sensitizer thiazolidinedione was correlated with a reduction of insulin resistance [36] and data with pioglitazone also suggest such a relationship [37]. Although a link between DS treatment and insulin sensitivity remains to be determined in the paradigm applied here, the present study argues for the potential of agents which like DS, increase insulin signaling and thereby generating a sustainable antidepressant-like effect at least as powerful as that of tricyclics.

## Competing interests

The authors declare that they have no competing interests.

## Authors' contributions

BC participated in the chronic stress experiment, statistical analysis and drafted the manuscript; HS participated in the design of the study and coordination; DM carried out RT-PCR; AR participated in the chronic stress experiment and tissue collection; GP participated in the design of the study and coordination; RC participated in the coordination of the study and helped to draft the manuscript; TS conceived of the study, and participated in its design and coordination and helped to draft the manuscript. All authors read and approved the final manuscript.
